# Hepatic ferredoxin reductase modulates mitochondrial function and iron homeostasis in metabolic dysfunction-associated steatotic liver disease

**DOI:** 10.21203/rs.3.rs-7014857/v1

**Published:** 2025-08-27

**Authors:** Tomoaki Tanaka, Ikki Sakuma, Rafael Gaspar, Panu Luukkonen, Brandon Hubbard, Daniel Vatner, Ali Nasiri, Sylvie Dufour, Mario Kahn, Mark Perelis, Yuki Taki, Akitoshi Nakayama, Masanori Fujimoto, Takashi Kono, Takashi Miki, Koutaro Yokote, Kitt Petersen, Varman Samuel, Gerald Shulman

**Affiliations:** Chiba University; Chiba University; Yale University; Yale School of Medicine; Yale University; Yale School of Medicine; Yale University School of Medicine; Yale School of Medicine; Yale School of Medicine; University of California San Diego; Chiba University; Chiba University; Chiba University; Chiba University; Chiba University; Chiba University; Yale Medical School; Yale School of Medicine; Yale School of Medicine

**Keywords:** IRP2, mitochondrial oxidation, oxidative stress, metabolic dysfunction-associated steatotic liver disease

## Abstract

Metabolic dysfunction-associated steatotic liver disease (MASLD) is the most common chronic liver disease globally. Disruptions in iron metabolism and mitochondrial oxidative function may cooperatively contribute to its pathogenesis. Ferredoxin reductase (FDXR), a mitochondrial flavoprotein, plays a critical role in mitochondrial respiratory supercomplex formation and iron-sulfur cluster biosynthesis—both essential for efficient oxidative metabolism. However, its role in MASLD remains unclear. Here, we knocked down hepatic Fdxr expression in the liver of C57BL/6 mice using N-acetyl galactosamineconjugated antisense oligonucleotides. [^13^C_5_]glutamine tracer infusions revealed that FDXR deficiency disrupted mitochondrial oxidative phosphorylation. In contrast, FDXR deficiency increased hepatic iron accumulation, reactive oxygen species, and lipid peroxidation. Mechanistically, FDXR deficiency disrupted iron-sulfur cluster assembly and reduced mitochondrial proteins such as succinate dehydrogenase complex iron-sulfur subunit B (SDHB), leading to mitochondrial dysfunction and steatosis. FDXR expression was upregulated in both human and murine MASLD livers, suggesting a compensatory protective response. Furthermore, hepatic overexpression of FDXR restored mitochondrial function, enhanced oxidative capacity, and ameliorated steatosis. These findings identify FDXR as a key regulator linking iron metabolism and mitochondrial integrity in MASLD and highlight its potential as a therapeutic target to prevent disease progression.

## Introduction

Metabolic dysfunction-associated steatotic liver disease (MASLD) is the most prevalent chronic liver disease worldwide and a major risk factor for hepatocellular carcinoma^1^. Recently, the U.S. Food and Drug Administration approved resmetirom, a β-selective thyroid hormone receptor agonist targeting the liver, for the treatment of metabolic dysfunction-associated steatohepatitis (MASH) with significant fibrosis. However, a Phase III clinical trial revealed that only 29% of participants responded to resmetirom, highlighting the urgent need for alternative therapeutic strategies and targets^2^.

Iron is a critical cofactor for numerous proteins involved in electron and oxygen transport, functioning either as part of iron-sulfur clusters or heme groups^3, 4, 5^. Disruptions in iron homeostasis have been linked to MASLD progression^6, 7, 8^, whereas iron depletion through phlebotomy reduces the progression of MASLD to advanced stages, such as MASH and liver fibrosis^7, 8, 9^. These findings suggest that regulating hepatic iron levels may offer a promising therapeutic strategy for MASLD management.

Given the importance of iron metabolism in MASLD, we focused on ferredoxin reductase (FDXR), a mitochondrial enzyme that initiates electron transfer to ferredoxin, facilitating heme and iron-sulfur cluster biogenesis^10, 11^. Mutations in FDXR impair iron-sulfur cluster function and lead to mitochondrial iron overload. This dysregulation increases reactive oxygen species (ROS), contributing to diseases such as auditory neuropathy and optic atrophy in humans^12, 13, 14^. In mice, Fdxr heterozygous knockout leads to hepatic steatosis, iron overload^15^, and increased susceptibility to hepatocellular carcinoma and hyperlipidemia^16^. These findings indicate that FDXR may influence MASLD pathogenesis through its roles in iron metabolism.

In addition to its metabolic functions, FDXR is also a known target of the tumor suppressor p53, a key regulator of cellular metabolism and mitochondrial function^17, 18, 19, 20^. Elevated hepatic p53 expression has been implicated in the progression of MASLD in human patients and animal models^21^. Furthermore, FDXR is critical for p53-dependent tumor suppression via iron regulatory protein 2^15^. Together, these findings suggest that FDXR contributed to MASLD pathogenesis by linking mitochondrial dysfunction, iron dysregulation, and oxidative stress. However, the exact contribution of FDXR to the regulation of mitochondrial function in MASLD remains unclear. We hypothesized that FDXR regulates MASLD progression by mediating hepatic mitochondrial oxidation, iron storage, and oxidative stress. To test this hypothesis, in the present study, we aimed to investigate how FDXR influences MASLD pathogenesis in vitro using liver-derived cells and in vivo in mice.

## Results

### p53-FDXR Pathway Activation in MASLD.

To investigate the role of p53–FDXR signaling in MASLD, we assessed the expression of *FDXR, p53*, and *p21* (a canonical p53 target gene) in liver samples from patients with nonalcoholic fatty liver or nonalcoholic steatohepatitis (NAFL/NASH; recently redefined as MASLD/MASH^22^), as well as from mice fed a choline-deficient, L-amino acid-defined, high-fat diet (CDAHFD), a widely used MASH mice model^23^.

**Figure 1:**
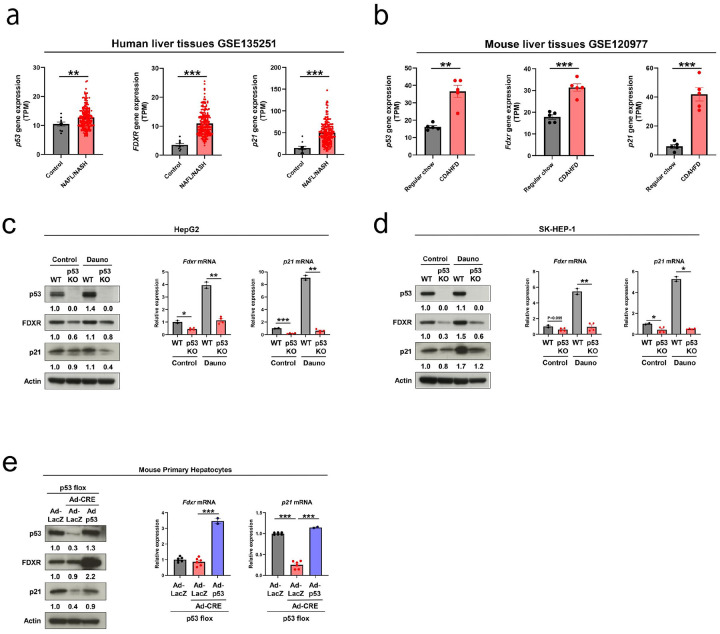
Hepatic expression of p53-FDXR correlates with nonalcoholic fatty liver disease in humans and a mouse model. **(a)** Expression of *p53*, *FDXR*, and *p21* in human liver samples quantified using Gene Expression Omnibus RNA-sequencing data (GSE135251 dataset); Control, n = 10; nonalcoholic fatty liver (NAFL)/nonalcoholic steatohepatitis (NASH), n = 206). **(b)** *p53*, *Fdxr*, and *p21* expression quantified in a metabolic dysfunction-associated steatohepatitis mouse model treated with a choline-deficient, L-amino acid-defined, high-fat diet using RNA-sequencing data from GSE120977. Control; regular chow, n = 5; CDAHFD, n = 5. *P < 0.05, **P < 0.01, ***P < 0.001 (Unpaired one-sided Student’s t-test). **(c)** Protein expression of p53, FDXR, and p21 assessed using immunoblotting analysis (*Left*), and mRNA expression of *FDXR* and *p21* assessed using qRT-PCR analysis (*Right*) in wild-type (WT) and p53-knockout (KO) HepG2 cells treated or untreated (control) with daunorubicin (Dauno; 1 μM) for 24 h. *P < 0.05, **P < 0.01, ***P < 0.001 (Unpaired one-sided Student’s t-test, WT versus p53-KO in control condition, WT versus p53 in daunorubicin treatment, respectively). (WT in control condition, n = 2; p53-KO in control condition, n = 4; WT in daunorubicin treatment, n = 2; p53-KO in daunorubicin treatment, n = 4). **(d)** Protein expression of p53, FDXR, and p21 assessed using immunoblotting analysis (*Left*) and mRNA expression of *FDXR* and *p21* assessed using qRT-PCR analysis (*Right*) in WT and p53-KO SK-HEP-1 cells. The cells were either treated or untreated (control) with daunorubicin (Dauno; 1 μM) for 24 h. Actin was used as an internal control in immunoblotting analysis. *P < 0.05, **P < 0.01, ***P < 0.001 (Unpaired one-sided Student’s t-test, WT versus p53-KO in control condition, WT versus p53 in daunorubicin treatment, respectively); WT in control condition, n = 2; p53-KO in control condition, n = 4; WT in daunorubicin treatment, n = 2; p53-KO in daunorubicin treatment, n = 4. **(e)** p53, FDXR, and p21 assessed using immunoblotting analysis (*Left*), and mRNA expression of *Fdxr* and *p21* assessed using qRT-PCR (*Right*) in primary hepatocytes derived from p53 flox mice treated with adenovirus expressing CRE recombinase deleted p53. *P < 0.05, **P < 0.01, ***P < 0.001 versus Ad-LacZ and Ad-CRE treatment (one-way analysis of variance [ANOVA] followed by Dunnett’s multiple comparison test). (Ad-LacZ, n = 5; Ad-LacZ and AdCRE, n = 5; Ad-LacZ, Ad-CRE, and Ad-p53, n = 2). All data in Figure 1 are presented as mean ± standard error of the mean (SEM).

Analysis of Gene Expression Omnibus (GEO) dataset revealed significantly higher expression of *FDXR, p53*, and *p21* in human NAFL/NASH livers than in controls (Fig. 1a). Similarly, CDAHFD-fed mice exhibited upregulation of *Fdxr, p53*, and *p21* in the liver compared to chow-fed controls (Fig. 1b).

### FDXR is Induced by p53 in Liver-Derived Cell Models.

To determine whether *FDXR* is directly regulated by p53 in liver cells, we used CRISPR/Cas9 to generate p53-knockout (KO) HepG2 and SK-HEP-1 cell lines, derived from human hepatocellular carcinoma (Fig. 1c, 1d). Under genotoxic stress induced by daunorubicin treatment, *FDXR* expression was significantly upregulated at both the mRNA and protein levels in a *p53*-dependent manner in wild-type HepG2 and SK-HEP-1 cells. However, this induction was absent in p53-KO cells (Fig. 1c, 1d).

Additionally, we assessed FDXR expression in primary hepatocytes derived from *p53* floxed mice, where adenoviral-mediated Cre recombinase expression efficiently knocked out *p53*. The findings showed that the depletion of p53 did not affect FDXR expression under basal conditions; however, adenoviralmediated p53 overexpression in *p53*-depleted hepatocytes markedly elevated *FDXR* expression (Fig. 1e).

### FDXR Forms Mitochondrial Supercomplexes and Regulates the Mitochondrial Respiratory Chain.

**Figure 2:**
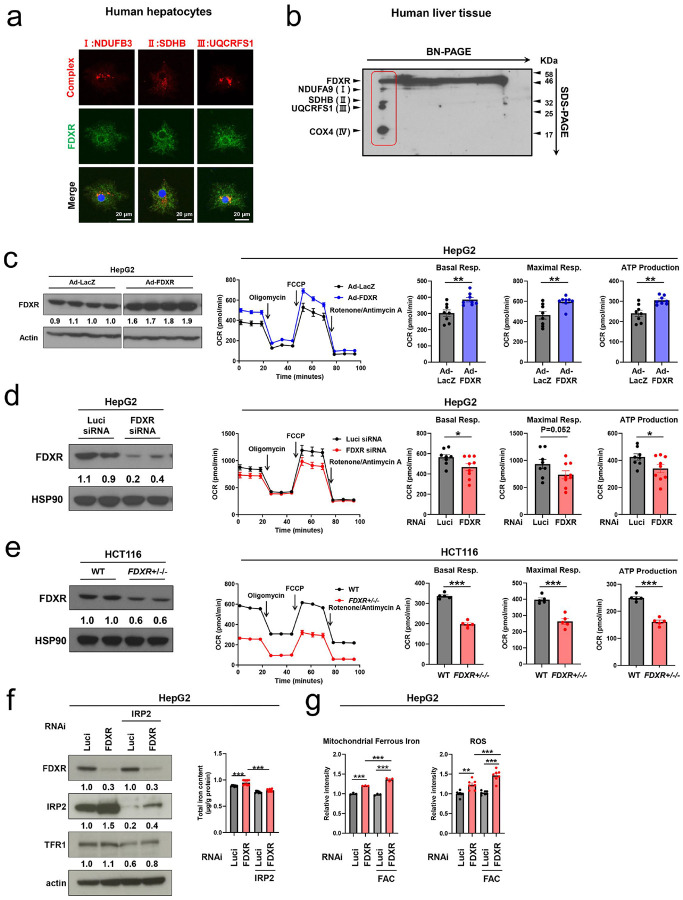
FDXR regulates the mitochondrial respiratory chain and cellular iron homeostasis. **(a)** Intracellular localization of FDXR and respiratory chain complexes (NDUFB3 for complex I, SDHB for complex II, and UQCRFS1 for complex III) using confocal microscopy in human hepatocytes. Nuclei were counterstained with DAPI. **(b)** Identification of mitochondrial supercomplexes and their association with FDXR in human liver tissue. The tissue was analyzed for mitochondrial supercomplexes using two-dimensional blue native-polyacrylamide gel electrophoresis (BN-PAGE) followed by sodium dodecyl sulfate-polyacrylamide gel electrophoresis (SDS-PAGE). Immunoblotting was performed with anti-FDXR, anti-NDUFA9, antiSDHB, anti-UQCRFS1, and anti-COX4 to detect complexes I, II, III, and IV, respectively. FDXR was detected in association with the mitochondrial supercomplexes (red-boxed area). **(c–e)** Oxygen consumption rates (OCRs) in HepG2 cells treated with adenovirus expressing FDXR (n = 7) or LacZ (n = 8) **(c)**, *FDXR* siRNA (n = 7) or Luci siRNA (n = 9) **(d)**, and in HCT116 cells with *FDXR*^+/−/−^ (n = 5) or wild-type (WT) (n = 5) **(e)**. OCR was measured at baseline and after treatment with oligomycin, FCCP, and a mixture of antimycin and rotenone. Immunoblotting was performed with anti-FDXR, anti-Actin and anti-HSP90 to confirm FDXR protein expression under these conditions. The middle lane in **(c)** has been removed for clarity; all shown lanes were run on the same gel and processed under identical conditions.” *P < 0.05, **P < 0.01, ***P < 0.001 (Unpaired one-sided Student’s t-test). **(f)** (Left) Immunoblots of HepG2 cell extracts showing expression of FDXR, IRP2, and TFR1 in HepG2 cells treated with respective siRNAs and (right) total intracellular iron measured using the QuantiChrom Iron Assay Kit in cells treated with FDXR or IRP2 siRNAs. *P < 0.05, **P < 0.01 (one-way analysis of variance [ANOVA] followed by Tukey’s multiple comparison test). **(g)** (Left) Mitochondrial ferrous iron levels measured using the Mito-FerroGreen assay and (right) reactive oxygen species (ROS) levels measured using the H2DCFDA assay in HepG2 cells under normal media and ferric ammonium citrate (FAC)-supplemented (100 μg/mL) media as iron loading. *P < 0.05, **P < 0.01, ***P < 0.001 (one-way ANOVA followed by Tukey’s multiple comparison test). All data in Figure 2 are presented as mean ± standard error of the mean (SEM).

To assess the involvement of FDXR in mitochondrial function, we first confirmed its localization in human hepatocytes using confocal microscopy. FDXR was partially colocalized with markers for respiratory chain complexes I (NDUFB3), II (SDHB), and III (UQCRFS1) (Fig. 2a).

Next, we investigated whether FDXR is associated with mitochondrial supercomplexes in liver tissue. Two-dimensional blue native sodium dodecyl sulfate-polyacrylamide gel electrophoresis (BN/SDS-PAGE) revealed colocalization of FDXR with mitochondrial supercomplexes comprising complexes I, II, III, and IV (Fig. 2b).

To evaluate the functional role of FDXR in mitochondrial respiration, we measured the oxygen consumption rate (OCR) in HepG2 and HCT116 cells under various experimental conditions. FDXR overexpression via adenovirus in HepG2 cells significantly increased basal respiration, maximal respiration, and ATP production compared to that in controls (Fig. 2c). Conversely, *FDXR* knockdown using small interfering RNA (siRNA) reduced these parameters (Fig. 2d). Similarly, HCT116 cells with chronic *FDXR* deficiency (*FDXR*^*+/−/−*^) exhibited substantial impairments in basal respiration, maximal respiration, and ATP production compared to that in wild-type cells (Fig. 2e).

### FDXR Regulates IRP2-Mediated Cellular Iron Homeostasis.

To investigate the role of FDXR in cellular iron homeostasis, we knocked down its expression in HepG2 cells using siRNA and analyzed its effects on iron-regulatory proteins (IRPs), which control intracellular iron levels^10, 24, 25^. *FDXR* knockdown markedly upregulated IRP2 levels, along with a slight upregulation of transferrin receptor 1 (TFR1) (Fig. 2f). Additionally, the intracellular total iron content was significantly elevated in *FDXR*-deficient cells. However, the co-knockdown of IRP2 abolished this effect, indicating that FDXR regulates iron levels through IRP2 (Fig. 2f).

To further investigate mitochondrial iron dynamics, we measured mitochondrial ferrous iron levels using the Mito-FerroGreen assay. *FDXR* knockdown increased mitochondrial ferrous iron levels under both normal media conditions and ferric ammonium citrate (FAC)-supplemented media (Fig. 2g, left panel). ROS levels were significantly elevated in FDXR-deficient HepG2 cells, particularly under FACsupplemented conditions (Fig. 2g, right panel).

### FDXR Deficiency Impairs Hepatic Tricarboxylic Acid (TCA) Cycle Flux and Mitochondrial Oxidative Metabolism.

**Figure 3:**
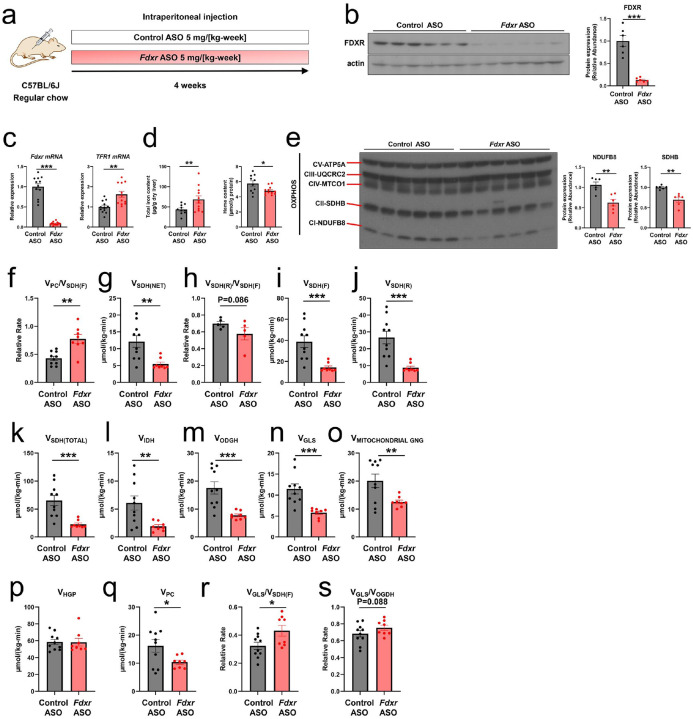
Fdxr antisense oligonucleotide suppresses FDXR expression and decreases mitochondrial tricarboxylic acid cycle flux in the liver. **(a)** Study design. Male C57BL6J mice were fed a regular chow diet and treated with 5 mg/kg/week of *Fdxr* antisense oligonucleotide (ASO) or control ASO for 4 weeks. **(b)** FDXR expression in the liver samples analyzed using immunoblotting (control ASO, n = 6, Fdxr ASO, n = 6). ***P < 0.001 (Unpaired one-sided Student’s t-test). **(c)** Hepatic *Fdxr* and *Tfr1* mRNA expression analyzed using qRT-PCR (control ASO n = 12, Fdxr ASO n = 12). **P < 0.01; ***P < 0.001 (Unpaired one-sided Student’s ttest). **(d)** Hepatic total iron and heme content analyzed using the QuantiChrom Iron and QuantiChrom Heme Assay Kits (control ASO, n = 10; Fdxr ASO; n = 12). *P < 0.05; **P < 0.01 (Unpaired one-sided Student’s t-test). **(e)** Immunoblot analysis of OXPHOS protein expression in whole liver lysates (control ASO, n = 6; Fdxr ASO, n = 6). **P < 0.01 (Unpaired one-sided Student’s t-test). **(f–t)** [^13^C_5_] glutamine-based flux analysis (Q-Flux) conducted in mice treated with Fdxr ASO (n = 8) or control ASO (n = 10). **(f)** Hepatic pyruvate carboxylase flux relative to succinate dehydrogenase forward flux (V_PC_/V_SDH(F)_). **(g)** Absolute rates of hepatic complex II/succinate dehydrogenase net flux (V_SDH(NET)_). **(h)** Rates of reverse succinate dehydrogenase flux relative to forward flux (V_SDH(R)_/V_SDH(F)_). **(i–k)** Absolute rates of succinate dehydrogenase forward flux (V_SDH(F)_) **(i)**, reverse flux (V_SDH(R)_) **(j)**, and total flux (V_SDH(TOTAL)_) **(k)**. **(l–o)** Absolute rates of isocitrate dehydrogenase flux (V_IDH_) **(l)**, α-ketoglutarate dehydrogenase flux (V_OGDH_) (*M*), glutaminase flux (V_GLS_) (N), and mitochondrial gluconeogenic carbon flux (V_MITOCHONDRIAL GNG_) **(o)**. **(p)** Hepatic glucose production (V_HGP_). **(q)** Absolute rates of V_PC_. **(r–s)** Hepatic glutaminase flux relative to succinate dehydrogenase forward flux (V_GLS_/V_SDH(F)_) **(r)** and hepatic glutaminase flux relative to αketoglutarate dehydrogenase flux (V_GLS_/V_OGDH_) **(s)**. *P < 0.05; **P < 0.01; ***P < 0.001 (Unpaired onesided Student’s t-test). All data in Figure 3 are presented as mean ± standard error of the mean (SEM).

To investigate the role of FDXR in vivo, C57BL6J mice were treated with *N*-acetyl galactosaminemodified antisense oligonucleotides (ASOs) to specifically knock down Fdxr in the liver (Fdxr ASO group). A separate control group received a nontargeting control ASO. Both groups were treated for 4 weeks while being maintained on a regular chow diet (Fig. 3a). *Fdxr* ASO group showed approximately 90% reduction in *Fdxr* mRNA and protein levels in the liver compared to that in the control ASO group (Fig. 3b, 3c). This knockdown was accompanied by increased *Tfr1* mRNA expression, indicating a compensatory upregulation of iron uptake via Tfr1 in response to hepatic iron deficiency. Consequently, hepatic total iron content was significantly elevated, whereas hepatic heme levels decreased in *Fdxr* ASO-treated mice (Fig. 3d). *Fdxr* ASO treatment reduced the abundance of SDHB protein, a key ironsulfur cluster-containing subunit of succinate dehydrogenase (SDH) (Fig. 3e). These findings suggest that *Fdxr* deficiency disrupts iron metabolism, potentially impairing mitochondrial function through altering the biosynthesis of heme and iron-sulfur clusters.

To evaluate the effect of *Fdxr* knockdown on hepatic mitochondrial function, we performed [^13^C_5_]glutamine-based flux analysis (Q-Flux)^26^ to assess TCA cycle activity. *Fdxr* ASO treatment significantly reduced succinate dehydrogenase (SDH) net flux (V_SDH(NET)_), pyruvate carboxylase (V_PC_), and mitochondrial fluxes that support gluconeogenesis (V_MITOCHONDRIAL GNG_) (Fig. 3f–o). Additionally, forward and reverse fluxes of SDH (V_SDH(F)_ and V_SDH(R)_) were reduced, leading to a 35% decrease in total SDH flux (Fig. 3h–k), indicating a significant impairment of TCA cycle activity. Furthermore, key fluxes associated with other mitochondrial enzymes, including isocitrate dehydrogenase (IDH) and oxoglutarate dehydrogenase (OGDH), were also markedly decreased (Fig. 3l–n). These reductions in mitochondrial enzymatic activity collectively led to the suppression of fluxes supporting gluconeogenesis (V_MITOCHONDRIAL GNG_, VPC) (Fig. 3o, 3q), although hepatic glucose production remained unchanged (Fig. 3p). Furthermore, increased V_GLS_/V_SDH(F)_ and V_GLS_/V_OGDH_ ratios in *Fdxr* ASO-treated mice showed a compensatory increase in glycolytic flux to meet energy demands under conditions of impaired mitochondrial function (Fig. 3r, 3s).

### Fdxr Overexpression Enhances TCA Cycle Flux in the Liver and Alleviates Steatosis.

To further investigate the role of *Fdxr* in hepatic mitochondrial function, we overexpressed it in the liver using a serotype 8 adeno-associated virus (AAV8) controlled by a thyroxine-binding globulin promoter (TBG). C57BL6J mice were injected with AAV8-TBG-FDXR (AAV-FDXR) or AAV8-TBG-EGFP (AAV-GFP) as a control (Supplementary Fig. 1a).

Immunoblot analysis confirmed the elevated expression of FDXR in the livers of AAV-FDXR-treated mice than that in those of the AAV-GFP-treated control mice (Supplementary, Fig. 1b). Next, we measured hepatic mitochondrial flux using positional isotopomer NMR tracer analysis (PINTA)^27^ to assess mitochondrial function (Supplementary Fig. 1c-1g). AAV-FDXR-treated mice exhibited increased citrate synthase flux (V_CS_) compared that in to control mice, indicating enhanced mitochondrial oxidative capacity (Supplementary Fig. 1f).

Additionally, the ratio of pyruvate carboxylase flux (V_PC_) to V_CS_ was reduced, suggesting improved mitochondrial efficiency (Supplementary Fig. 1d). Hepatic glucose production and V_PC_ rates were also slightly elevated in AAV-FDXR-treated mice (Supplementary Fig. 1c, 1e). Furthermore, hepatic triglyceride content decreased in AAV-FDXR-treated mice (Supplementary Fig. 1g).

### Choline-deficient L-amino acid-defined high-fat diet acutely induces hepatic lipid accumulation and inflammation followed by p53-FDXR Pathway Activation.

Mice develop a steatohepatitis phenotype (steatosis and inflammation) within one week of CDAHFD feeding^28^. To evaluate the hepatic transcriptomic changes associated with one-week CDAHFD-induced steatohepatitis, RNA-seq analysis was conducted. CDAHFD was administered for one week (Supplementary Fig. 2a), regular chow fed mice were used as a control.

Analysis of differentially expressed genes (DEGs) revealed 734 upregulated and 83 downregulated genes in the CDAHFD group compared to regular chow group (Supplementary Fig. 2b). Gene set enrichment analysis (GSEA) demonstrated that CDAHFD group exhibited significant enrichment in inflammatory pathways, including “TNFα signaling via NFκB” and “inflammatory response.” Additionally, lipid-related pathways such as “fatty acid metabolism” and “cholesterol homeostasis” were upregulated, reflecting the metabolic disturbances characteristic of steatohepatitis (Supplementary Fig. 2c). Notably, enrichment of the “p53 pathway” was also observed. In accordance with this, we confirmed the upregulation of *p53, Fdxr*, and *p21* expression levels (Supplementary Fig. 2d).

### Fdxr Deficiency Exacerbates Hepatic Steatosis and Oxidative Stress.

**Figure 4:**
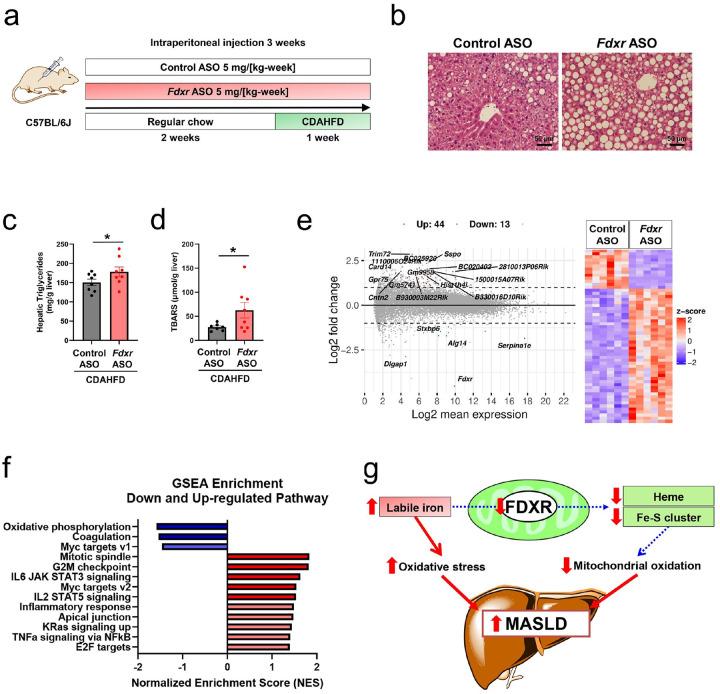
FDXR deficiency aggravates steatosis and oxidative stress in the liver. **(a)** Study design. Control antisense oligonucleotide (ASO) or *Fdxr* ASO was administered to C57BL6J mice for 3 weeks. Mice were fed regular chow for 2 weeks, followed by a choline-deficient, L-amino acid-defined, high-fat diet (CDAHFD) for 1 week. **(b)** Hematoxylin and eosin staining of liver sections from *Fdxr* ASO- and control ASO-treated mice. **(c)** Hepatic triglyceride content in *Fdxr* ASO- and control ASO-treated mice; control ASO (n = 8), *Fdxr* ASO (n = 8). *P < 0.05 (Unpaired one-sided Student’s t-test). **(d)** Levels of hepatic thiobarbituric acid-reactive substances (TBARS) in *Fdxr* ASO- and control ASO-treated mice; control ASO (n = 7), *Fdxr* ASO (n = 8). *P < 0.05 (Unpaired one-sided Student’s t-test). Transcriptomic changes based on RNA-seq analysis of hepatic *Fdxr* knockdown in CDAHFD-fed mice are shown in **(e)** and **(f)**. **(e)**MA plot showing differentially expressed genes (DEGs) between *Fdxr* ASO and control ASO groups. **(f)**Gene set enrichment analysis (GSEA) illustrating pathway enrichment based on DEGs in the *Fdxr* ASO group. **(g)** The role of FDXR in metabolic dysfunction-associated steatotic liver disease (MASLD). FDXR deficiency leads to mitochondrial dysfunction and increased hepatic labile iron, followed by oxidative stress and MASLD progression.

To determine whether FDXR activation is a protective response or a harmful response to CDAHFD induced steatohepatitis, we treated mice with Fdxr ASO or control ASO, we treated mice with *Fdxr* ASO or control ASO for 3 weeks, followed by 1 week on a CDAHFD (Fig. 4a). Histological analysis of liver sections revealed that *Fdxr* ASO treatment exacerbated CDAHFD-induced lipid droplet accumulation compared to control ASO treatment (Fig. 4b). Consistently, hepatic triglyceride content was significantly increased in *Fdxr* ASO-treated mice (Fig. 4c).

We measured hepatic thiobarbituric acid-reactive substances (TBARS), a marker of lipid peroxidation, to assess oxidative stress. TBARS levels were significantly elevated in *Fdxr* ASO-treated mice compared to those in control mice, indicating increased oxidative damage (Fig. 4d).

Analysis of differentially expressed genes (DEGs) from RNA sequencing identified 44 upregulated and 13 downregulated genes in the *Fdxr* ASO group compared to the control ASO group (Fig. 4e). Gene set enrichment analysis (GSEA) revealed significant enrichment of inflammatory pathways in the *Fdxr* ASO group, including “TNFα signaling via NFκB” and “inflammatory response,” indicating that *Fdxr* knockdown exacerbates CDAHFD-induced hepatic inflammation. In contrast, the oxidative phosphorylation pathway was significantly downregulated, suggesting impaired mitochondrial oxidative metabolism resulting from FDXR deficiency (Fig. 4f).

## Discussion

In this study, we investigated the role of FDXR in regulating mitochondrial function and iron metabolism in the liver, with a focus on its contribution to the pathogenesis of MASLD. Our findings demonstrate that *FDXR* deficiency impairs mitochondrial oxidation, disrupts iron homeostasis, and exacerbates oxidative stress, thereby promoting steatosis. Conversely, hepatic overexpression of *FDXR* enhanced mitochondrial oxidative capacity and alleviates steatosis, providing insight into its potential as a therapeutic target for MASLD.

Previous studies have shown that p53 upregulates FDXR in response to cellular stress, particularly in the context of colorectal cancer, where this upregulation sensitizes cells to apoptosis^17, 18^. Consistent with these findings, our study showed p53-dependent induction of FDXR in liver cells under stress conditions, suggesting a protective role of this pathway in liver disease. Moreover, our analysis of human MASLD/MASH liver samples and a murine MASH model revealed elevated hepatic expression of FDXR, p53, and p21, indicating that p53-FDXR signaling is activated during MASLD progression. This activation likely reflects a protective response to cellular damage and inflammation associated with the disease.

One of the key findings of this study is the dual role of FDXR in mitochondrial function and iron metabolism. FDXR is essential for the biogenesis of iron-sulfur clusters and heme, which are critical for maintaining the mitochondrial respiratory chain. In this study, FDXR deficiency reduced SDHB expression, an iron-sulfur cluster subunit of SDH, leading to impaired TCA cycle flux and mitochondrial metabolism. Hepatic steatosis develops when there is an imbalance between hepatic lipid synthesis (import of triglyceride rich lipoprotein remnants, uptake of circulating fatty acids, de novo biosynthesis) and hepatic lipid disposal (lipid oxidation, VLDL assembly and export, bile acid synthesis and export). Thus, the impairment in mitochondrial oxidative fluxes seen in Fdxr knockdown mice likely contributes to increased hepatic steatosis in these mice. In contrast, FDXR overexpression enhanced TCA cycle flux in the liver and alleviates steatosis. These findings are consistent with recent studies demonstrating that liver-targeted mitochondrial protonophores promote increased rates of hepatic mitochondrial fat oxidation leading to reductions in hepatic triacylglycerol and diacylglycerol content^29, 30, 31, 32, 33^.

A previous study demonstrated that impaired CPT1A expression is associated with disrupted fatty acid oxidation in FDXR-depleted cancer cells^34^. In particular, reduced CPT1A expression has been reported in FDXR-deficient contexts, suggesting a potential link between FDXR and mitochondrial fatty acid transport. While our current findings do not establish a direct relationship between FDXR knockdown and fatty acid oxidation, it is plausible that impaired mitochondrial function and reduced TCA cycle flux may secondarily influence lipid catabolism. Future studies will be necessary to clarify whether FDXR modulates fatty acid oxidation through regulation of CPT1A or other components of the fatty acid oxidation pathway.

Additionally, FDXR deficiency elevated TFR1 expression, resulting in excessive hepatic iron accumulation. Excess ferrous iron promotes ROS production through Fenton chemistry, further exacerbating oxidative stress followed by inflammation. These results are consistent with previous studies implicating FDXR in iron regulation and oxidative stress, particularly in human fibroblast models^13^. Moreover, a recent study demonstrated that FDXR deficiency in mice leads to mitochondrial iron overload and increased ROS production in brain tissue, which was associated with neurodegeneration and suggested neuroinflammation^35^. While their work primarily focused on neurodegeneration, the presence of hepatic iron deposition and oxidative stress supports the possibility that FDXR deficiency may trigger hepatic inflammation through similar iron-mediated oxidative mechanisms.

In summary, our findings highlight the interplay between mitochondrial dysfunction, iron metabolism, and oxidative stress in the progression of MASLD. In the early stages of MASLD, increased mitochondrial oxidative function may serve as an adaptive response to elevated substrate availability and ATP demand. However, as the disease advances to MASH, this adaptive response diminishes, resulting in impaired mitochondrial oxidative capacity, which exacerbates steatosis and inflammation^36^. Our data demonstrates that FDXR deficiency accelerates this transition by impairing mitochondrial flux and promoting oxidative damage (Fig. 4g). In contrast, hepatic overexpression of FDXR restored mitochondrial oxidation, increased TCA cycle activity, and alleviated steatosis in a mouse model, suggesting that therapeutic strategies aimed at enhancing FDXR activity could have significant potential in addressing MASLD. Restoring mitochondrial function and re-establishing iron homeostasis through FDXR-targeted interventions may help slow or reverse disease progression.

Supporting this concept, systemic delivery of AAV-Fdxr has been shown to ameliorate mitochondrial dysfunction, iron overload, and neuroinflammation in Fdxr mutant mice, providing proof-of-principle for gene therapy-based rescue of FDXR-deficient phenotypes^37^. While this approach utilized a broadly tropic AAV-PHP.B vector, future strategies employing hepatocyte-targeted vectors, such as AAV8 along with the thyroxine-binding globulin (TBG) promoter, may enable liver-specific restoration of FDXR function. AAV8-based liver-directed gene therapy has already shown clinical utility, as demonstrated by the Phase 1/2 trial of DTX401 in adults with glycogen storage disease type Ia, in which hepatic delivery of a G6PC1 transgene via AAV8 resulted in sustained transgene expression, and improved metabolic control, without any serious treatment-related adverse events^38^. This approach also holds promise for therapeutic application in MASLD, a liver-predominant disorder characterized by mitochondrial and iron-related dysfunction.

## Methods

### Analysis of GEO Data.

GSE135251^39^ and GSE120977^40^ datasets were used to assess the expression of hepatic FDXR, p53, and p21 in human and mouse liver samples, respectively. RNA-seq data were analyzed using the GEO RNAseq Experiments Interactive Navigator (GREIN) platform, which provides gene expression values in transcripts per million (TPM). To validate the clinical relevance of our experimental findings in human MASLD, we analyzed publicly available RNA-sequencing data from the GSE135251 dataset. In parallel, we utilized the GSE120977 dataset to examine changes in FDXR expression in a CDAHFD-induced MASH mouse model.

### Cell Lines and Cell Culture.

Human hepatocarcinoma (HepG2 and SK-HEP-1) and human colon carcinoma (HCT116) cell lines were cultured in Dulbecco’s Modified Eagle’s Medium (DMEM) supplemented with 10% fetal bovine serum (FBS) and antibiotics.

### Quantitative Reverse Transcriptase PCR (qRT-PCR).

qRT-PCR was performed as described previously (39). Briefly, total RNA was extracted from cells using the RNeasy Kit (Qiagen, Valencia, CA, USA). Four micrograms of RNA were reverse transcribed using the ReverTra Ace qPCR RT Kit (Toyobo, Tokyo, Japan). The resulting cDNA was analyzed via qPCR using the 7500 Fast Real-Time PCR System (Applied Biosystems, Foster City, CA, USA) with primers listed in Supplementary Table 1.

### Immunoblot Analysis.

Western blotting was performed using established protocols (40). The following antibodies were used: FDXR (Santa Cruz Biotechnology), p53 DO1 (Santa Cruz Biotechnology), p21WAF1 (Calbiochem), actin (Sigma), NDUFV1 (Abnova), NDUFA9 (Abcam), Complex II (Invitrogen), SDHB (Abcam), UQCRFS1 (Abcam), COX4 (Cell Signaling Technology), IRP2 (Abcam), TFR1 (Santa Cruz Biotechnology), HSP90 (Cell Signaling Technology), and OXPHOS (Abcam).

### RNA Interference.

The siRNA oligonucleotides targeting *FDXR*, *IRP2*, or *luciferase* were synthesized by Qiagen, Valencia, CA, USA. The target sequences were as follows: *FDXR* siRNA, 5′-CCACGCTGTGGTGCTGAGCTA-3′; *IRP2* siRNA, 5′-CTGCGTGTTAAACCTTATATA-3′; luciferase siRNA, 5′-AACTTACGCTGAGTACTTCGA − 3′. HepG2 cells were transfected with the respective siRNA using Lipofectamine RNAiMAX (Invitrogen). Cells were analyzed 48 hours after transfection.

### Immunofluorescence Analysis.

Human hepatocytes were cultured in 4-well chamber slides at a density of 2 × 10^4^ cells per well. The cells were fixed in absolute ethanol at − 20°C for 10 min, followed by incubation with a blocking solution and primary antibodies targeting FDXR (Santa Cruz Biotechnology), NDUFB3 (Abcam), SDHB (Abcam), and UQCRFS1 (Abcam). Fluorescence images were captured using a confocal laser scanning microscope (LSM Meta, Zeiss, Germany).

### Preparation of Mitochondrial Fractions.

Human liver tissue samples were washed in ice-cold phosphate-buffered saline (PBS) free of Ca^2+^ and Mg^2+^, then gently resuspended in 500 μL of sucrose buffer containing 0.07 M sucrose, 10 mM HEPES (pH 7.4), 0.1 mM EDTA, and 0.22 M mannitol. The tissue was homogenized on ice using a Dounce tissue homogenizer. The homogenate was centrifuged at 500 × g for 15 min at 4°C to remove unlysed cells and nuclei. The resulting supernatant was further centrifuged at 10,000 × g for 15 min at 4°C to isolate the mitochondrial fraction, which was resuspended in 500 μL of sucrose buffer and stored at − 80°C. Protein concentration was measured using the BCA Protein Assay Kit (Thermo Fisher Scientific Inc., Waltham, MA, USA).

### BN/SDS-PAGE.

The mitochondrial pellet (50 μg) was resuspended in 10 μL of buffer containing 50 mM bisTris (pH 7.0), 1 M 6-aminocaproic acid, and 2% dodecyl-D-maltoside. After 20 min of incubation at 4°C, solubilized proteins were separated via centrifugation at 22,000 × g. The supernatant containing solubilized proteins was mixed with 0.5 μL of sample buffer (5% G-250 in 0.5 M 6-aminocaproic acid). Two-dimensional BN/SDS-PAGE was performed. The first-dimension BN-PAGE (8–12%) was followed by the seconddimension SDS-PAGE (10%). The cathode buffer comprised 50 mM Tricine, 15 mM bisTris (pH 7.0), and 0.02% G-250, while the anode buffer contained 50 mM bisTris (pH 7.0). For the first dimension, electrophoresis was conducted on ice at 100 V for 10 min, then 200 V for 20 min, and finally 500 V for 60 min. Strips from the first-dimension gel were excised and subjected to second-dimension SDS-PAGE, performed on ice under conditions described previously^41^.

### Measurement of Oxygen Consumption (OCRs).

Oxygen consumption analysis was performed as described previously (42). HepG2 and HCT116 cells were seeded in a Seahorse XF24 cell culture plate (Seahorse Bioscience). Cells were washed with XF24 Assay Media, prewarmed to approximately 37°C, and 525 μL of the assay media was added to each well. Before measurements, the cells in assay media were incubated in an unbuffered, humidified incubator at 37°C for 1 h to equilibrate temperature and pH. OCRs were then measured using the Seahorse XF Analyzer.

### Measurement of Total Iron Content in Cells.

Total iron content in cells was quantified using the QuantiChrom Iron Assay Kit (BioAssay Systems) according to the manufacturer’s instructions. Briefly, cells were lysed in a buffer suitable for protein and metal extraction. The lysates were centrifuged to remove debris, and the supernatant was collected. For the assay, 50 μL of the lysate was mixed with the reagents provided in the kit protocol. Absorbance at 590 nm was measured using a microplate reader. Total iron content was calculated using a standard curve prepared with the kit’s iron standards. Results were normalized to protein concentration, determined using the BCA Protein Assay Kit (Thermo Fisher Scientific Inc.), to standardize iron levels relative to cellular mass and ensure comparability between samples.

### Measurement of Mitochondrial Ferrous Iron Content.

Mitochondrial ferrous iron levels were assessed using the Mito-FerroGreen Assay Kit (Dojindo) following the manufacturer’s instructions. Briefly, HepG2 cells were cultured in 6-well plates under standard conditions. After treatment with siRNA or FAC supplementation as specified, cells were washed with PBS and incubated with the Mito-FerroGreen reagent diluted in assay buffer.

Cells were incubated at 37°C for 30 min in the dark, and fluorescence intensity was measured using a microplate reader with excitation and emission wavelengths of 488 nm and 530 nm, respectively. The fluorescence intensity was normalized to protein concentration, determined using the BCA Protein Assay Kit (Thermo Fisher Scientific Inc).

### Measurement of ROS.

ROS levels were quantified using 2’,7’-dichlorodihydrofluorescein diacetate (H2DCFDA; Molecular Probes) following the manufacturer’s instructions. HepG2 cells were cultured in 6-well plates and treated according to the experimental setup. After treatment, cells were washed twice with PBS and incubated with 10 μM H2DCFDA diluted in PBS for 30 min at 37°C in the dark. Following incubation, cells were washed again with PBS to remove excess dye, and fluorescence intensity was measured using a microplate reader with excitation and emission wavelengths of 485 nm and 535 nm, respectively. ROS levels were normalized to the total protein concentration of each sample, determined using the BCA Protein Assay Kit (Thermo).

### Generation of Adenovirus.

The coding sequence of human FDXR was cloned into the pENTR4 vector and subsequently recombined into the Gateway-based pAd/CMV/V5-DEST vector (Invitrogen). Recombinant adenovirus was amplified in HEK 293A cells. Recombinant adenoviruses containing LacZ, Cre recombinase, and p53 were also generated in a similar manner.

### Generation of p53 Knockout HepG2 and SK-HEP-1 Cells.

p53 knockout HepG2 and SK-HEP-1 cells were established as described previously (40) using the CRISPR/Cas9 system based on the protocol by Cong et al. (43). The backbone vector pX459 pSpCas9(BB)-2A-Puro was obtained from Addgene (Massachusetts, USA). To clone the target guide RNA sequence for p53 into the pX459 backbone, oligos were synthesized by Eurofins Genomics (Tokyo, Japan), annealed, and phosphorylated using T4 DNA Ligase Reaction Buffer and T4 Polynucleotide Kinase (New England Biolabs, Massachusetts, USA) at 37°C for 30 min, followed by 95°C for 5 min. The pX459 plasmid was digested with BbsI (Thermo Scientific, Massachusetts, USA) at 37°C for 30 min and gel-purified using the QIAquick Gel Extraction Kit (QIAGEN, Hilden, Germany). The annealed oligos were ligated into pX459 at room temperature for 10 min using the Quick Ligation Kit (New England Biolabs) and purified with PlasmidSafe exonuclease (Cambio, Cambridge, UK) at 37°C for 30 min. The plasmids were transformed into Stbl3 cells, and appropriate clones were amplified and purified using NucleoBond Xtra Midi (Takara, Kusatsu, Japan). The pX459-p53 guide RNA plasmid was transfected into HepG2 or SK-HEP-1 cells using Lipofectamine 3000 (Thermo Scientific) according to the manufacturer’s instructions. Transfected HepG2 and SK-HEP-1 cells were selected with puromycin (Wako, Osaka, Japan), and monoclonal cell lines were established using the limiting dilution method.

### Animal Studies.

Nine-week-old male C57BL6J mice were obtained from Charles River Laboratories (Wilmington, MA) and acclimated for at least 3 days before the experiment. Mice were housed under a 12-h light/dark condition and provided access to food and water ad libitum. Diets included standard chow (Envigo 2108S, Envigo, Madison, WI, USA), a CDAHFD (A06071302, Research Diets, New Brunswick, NJ, USA), or a high-fat diet (D12492, Research Diets). Mice received weekly intraperitoneal injections of GalNAcmodified chimeric ASOs targeting Fdxr or a nontargeting control ASO at a dose of 5 mg/kg. Animals were sacrificed under isoflurane or pentobarbital anesthesia. All procedures were approved by the Institutional Animal Care and Use Committee of Yale University.

### Tracer-Based Flux Studies.

Hepatic mitochondrial flux was assessed using Q-Flux and PINTA methodologies, as described previously^26, 27, 29, 42^. For the Q-Flux study, briefly, following an overnight fast, mice under gentle tail restraint underwent a primed-continuous infusion of either [¹³C_5_] glutamine (6 mmol/kg·min) or [¹³C_4_] aspartate (10 mmol/kg·min), along with [3-^³^H] glucose (0.1 mCi/min), for 2 hours. Blood samples were collected via tail vein at 120 minutes for the measurement of plasma glucose and whole-body hepatic glucose production (HGP). All mice were anesthetized with an intravenous injection of sodium pentobarbital (150 mg/kg), and tissues were rapidly collected and snap-frozen in liquid nitrogen. For the PINTA study, briefly, following an overnight fast, mice under gentle tail restraint underwent a primedcontinuous infusion of [3-^³^H]glucose (PerkinElmer) at a rate of 0.044 μCi/min and [3-^¹³^C]sodium lactate (Cambridge Isotopes) at a rate of 30 μmol/kg·min for a total duration of 120 minutes to measure V_HGP_, V_PC/VCS_, V_PC_, and V_CS_.

### Histological Analysis.

Mouse liver tissues were fixed in 10% (w/v) neutral buffered formalin, processed, and embedded into paraffin blocks. Tissue sections were stained with hematoxylin and eosin by Yale Pathology Tissue Services for histological analysis.

### Heme and Iron Assay of Liver Tissues.

Heme and total iron levels in liver tissues were measured using the QuantiChrom Heme Assay Kit and QuantiChrom Iron Assay Kit (BioAssay Systems) according to the manufacturer’s instructions. Approximately 200 mg liver tissue was homogenized in 5 mL distilled water. The protein concentration of the homogenate was determined, and the sample was diluted to approximately 5 mg protein/mL. The homogenate was centrifuged at 2,000 × g for 5 min, and 50 μL of the supernatant was used to determine heme levels following the DIHM-250 kit instructions. Heme content was reported as nmol/mg protein. For iron measurement, liver tissue samples were dried overnight at 106°C and weighed. Samples were solubilized in 6 N nitric acid by heating at 100°C to release protein-associated iron. The solution was neutralized with NaOH, diluted in deionized water, and assayed for iron concentration using the DIFE-250 kit. Absorbance at 400 nm (heme) and 590 nm (iron) was measured using a microplate reader.

### TBARS Assay.

Liver tissue TBARS levels were measured using the TBARS Assay Kit (Cayman Chemical) according to the manufacturer’s instructions. Approximately 50 mg liver tissue was homogenized in the provided assay buffer using a tissue homogenizer. The homogenate was centrifuged at 1,600 × g for 10 min at 4°C, and the supernatant was collected. A 100 μL sample was mixed with 100 μL SDS solution and 4 mL thiobarbituric acid reagent. The mixture was heated at 95°C for 1 h, cooled on ice, and centrifuged at 1,600 × g for 10 min. The supernatant was transferred to a microplate, and absorbance at 532 nm was measured using a microplate reader. TBARS levels were calculated using malondialdehyde (MDA) standard curve and expressed as nmol MDA/mg protein, representing lipid peroxidation.

### Liver Triglyceride Measurement.

Hepatic triglyceride content was assessed using a modified Folch method followed by enzymatic quantification^28, 43^. Approximately 50 mg liver tissue was homogenized in a chloroform/methanol mixture (2:1, v/v) to extract lipids. The homogenate was vortexed thoroughly and centrifuged at 1,000 × g for 10 min to separate the phases. The organic phase was carefully transferred to a new tube, dried under nitrogen gas, and resuspended in isopropanol. Triglyceride content in the extracted lipid fraction was quantified using the Triglyceride-SL Reagent (Sekisui Diagnostics, USA) following the manufacturer’s instructions. Absorbance was measured at 550 nm using a microplate reader. Hepatic triglyceride content was calculated using a standard curve of a glycerol standard and expressed as mg triglycerides/g liver tissue.

### RNA Sequencing and Transcriptomic Analysis

Total RNA was extracted from frozen mouse liver tissues using the RNeasy Mini Kit (Qiagen), according to the manufacturer’s instructions. RNA quantity and quality were assessed using a NanoDrop spectrophotometer and Agilent 2100 Bioanalyzer. Samples with an RNA integrity number (RIN) > 7.0 were used for library preparation. RNA-seq libraries were prepared using the QuantSeq 3′ mRNA-Seq Library Prep Kit (Lexogen, Vienna, Austria), following the manufacturer’s protocol. Sequencing was performed on an Illumina NextSeq 500 platform (Illumina, San Diego, CA). Raw sequencing reads were uploaded to RNA-chef (https://rnachef.org), a cloud-based pipeline for automated RNA-seq data processing and analysis. Reads were aligned to the mouse reference genome (GRCm38/mm10) using STAR, and gene-level counts were obtained using feature Counts. Differential gene expression analysis was performed using DESeq2 within RNA-chef, with differentially expressed genes (DEGs) defined as those with a fold change ≥ 2 or ≤ 0.5 and a false discovery rate (FDR) < 0.05. Gene set enrichment analysis (GSEA) was conducted using the Molecular Signatures Database (MSigDB) Hallmark gene sets to identify biological pathways enriched in the CDAHFD group compared to the control group. Visualization and data interpretation were performed using RNA-chef’s integrated analysis tools.

## Supplementary Material

Supplementary Files

This is a list of supplementary files associated with this preprint. Click to download.

- FDXRsuppleTable05122025.docx
- FDXRSupfigureswithlegends0628.pdf

## References

[1] Loomba R, Friedman SL, Shulman GI. Mechanisms and disease consequences of nonalcoholic fatty liver disease. Cell 184, 2537–2564 (2021). 33989548 10.1016/j.cell.2021.04.015 PMC12168897

[2] Harrison SA, A Phase 3, Randomized, Controlled Trial of Resmetirom in NASH with Liver Fibrosis. The New England journal of medicine 390, 497–509 (2024). 38324483 10.1056/NEJMoa2309000

[3] Lill R. Function and biogenesis of iron-sulphur proteins. Nature 460, 831–838 (2009). 19675643 10.1038/nature08301

[4] Sheftel A, Stehling O, Lill R. Iron-sulfur proteins in health and disease. Trends in endocrinology and metabolism: TEM 21, 302–314 (2010). 20060739 10.1016/j.tem.2009.12.006

[5] Lill R, The role of mitochondria in cellular iron-sulfur protein biogenesis and iron metabolism. Biochimica et biophysica acta 1823, 1491–1508 (2012). 22609301 10.1016/j.bbamcr.2012.05.009

[6] Simcox JA, McClain DA. Iron and diabetes risk. Cell metabolism 17, 329–341 (2013). 23473030 10.1016/j.cmet.2013.02.007 PMC3648340

[7] Dongiovanni P, Fracanzani AL, Fargion S, Valenti L. Iron in fatty liver and in the metabolic syndrome: a promising therapeutic target. Journal of hepatology 55, 920–932 (2011). 21718726 10.1016/j.jhep.2011.05.008

[8] Nelson JE, Klintworth H, Kowdley KV. Iron metabolism in Nonalcoholic Fatty Liver Disease. Current gastroenterology reports 14, 8–16 (2012). 22124850 10.1007/s11894-011-0234-4

[9] Tajima S, Iron reduction by deferoxamine leads to amelioration of adiposity via the regulation of oxidative stress and inflammation in obese and type 2 diabetes KKAy mice. American journal of physiology Endocrinology and metabolism 302, E77–86 (2012). 21917632 10.1152/ajpendo.00033.2011

[10] Shi Y, Ghosh M, Kovtunovych G, Crooks DR, Rouault TA. Both human ferredoxins 1 and 2 and ferredoxin reductase are important for iron-sulfur cluster biogenesis. Biochimica et biophysica acta 1823, 484–492 (2012). 22101253 10.1016/j.bbamcr.2011.11.002 PMC3546607

[11] Sheftel AD, Humans possess two mitochondrial ferredoxins, Fdx1 and Fdx2, with distinct roles in steroidogenesis, heme, and Fe/S cluster biosynthesis. Proceedings of the National Academy of Sciences of the United States of America 107, 11775–11780 (2010). 20547883 10.1073/pnas.1004250107 PMC2900682

[12] Peng Y, Biallelic mutations in the ferredoxin reductase gene cause novel mitochondriopathy with optic atrophy. Human molecular genetics 26, 4937–4950 (2017). 29040572 10.1093/hmg/ddx377 PMC5886230

[13] Paul A, FDXR Mutations Cause Sensorial Neuropathies and Expand the Spectrum of Mitochondrial Fe-S-Synthesis Diseases. American journal of human genetics 101, 630–637 (2017). 28965846 10.1016/j.ajhg.2017.09.007 PMC5630197

[14] Wang J, Biallelic FDXR mutations induce ferroptosis in a rare mitochondrial disease with ataxia. Free Radic Biol Med 230, 248–262 (2025). 39954867 10.1016/j.freeradbiomed.2025.02.012

[15] Zhang Y, Ferredoxin reductase is critical for p53-dependent tumor suppression via iron regulatory protein 2. Genes & development 31, 1243–1256 (2017). 28747430 10.1101/gad.299388.117 PMC5558926

[16] Zhang Y, Mohibi S, Vasilatis DM, Chen M, Zhang J, Chen X. Ferredoxin reductase and p53 are necessary for lipid homeostasis and tumor suppression through the ABCA1-SREBP pathway. Oncogene, (2022). 10.1038/s41388-021-02100-0 PMC8933276 35121827

[17] Hwang PM, Ferredoxin reductase affects p53-dependent, 5-fluorouracil-induced apoptosis in colorectal cancer cells. Nature medicine 7, 1111–1117 (2001). 10.1038/nm1001-1111 PMC4086305 11590433

[18] Liu G, Chen X. The ferredoxin reductase gene is regulated by the p53 family and sensitizes cells to oxidative stress-induced apoptosis. Oncogene 21, 7195–7204 (2002). 12370809 10.1038/sj.onc.1205862

[19] Nagano H, p53-inducible DPYSL4 associates with mitochondrial supercomplexes and regulates energy metabolism in adipocytes and cancer cells. Proceedings of the National Academy of Sciences of the United States of America 115, 8370–8375 (2018). 30061407 10.1073/pnas.1804243115 PMC6099896

[20] Suzuki S, Phosphate-activated glutaminase (GLS2), a p53-inducible regulator of glutamine metabolism and reactive oxygen species. Proceedings of the National Academy of Sciences of the United States of America 107, 7461–7466 (2010). 20351271 10.1073/pnas.1002459107 PMC2867754

[21] Tomita K, p53/p66Shc-mediated signaling contributes to the progression of non-alcoholic steatohepatitis in humans and mice. Journal of hepatology 57, 837–843 (2012). 22641095 10.1016/j.jhep.2012.05.013

[22] Rinella ME, A multisociety Delphi consensus statement on new fatty liver disease nomenclature. Journal of hepatology 79, 1542–1556 (2023). 37364790 10.1016/j.jhep.2023.06.003

[23] Gallage S, A researcher’s guide to preclinical mouse NASH models. Nat Metab 4, 1632–1649 (2022). 36539621 10.1038/s42255-022-00700-y

[24] Moroishi T, Nishiyama M, Takeda Y, Iwai K, Nakayama KI. The FBXL5-IRP2 axis is integral to control of iron metabolism in vivo. Cell metabolism 14, 339–351 (2011). 21907140 10.1016/j.cmet.2011.07.011

[25] Zhang J, Chen X. p53 tumor suppressor and iron homeostasis. The FEBS journal 286, 620–629 (2019). 30133149 10.1111/febs.14638 PMC6379103

[26] Hubbard BT, Q-Flux: A method to assess hepatic mitochondrial succinate dehydrogenase, methylmalonyl-CoA mutase, and glutaminase fluxes in vivo. Cell metabolism 35, 212–226.e214 (2023). 36516861 10.1016/j.cmet.2022.11.011 PMC9887731

[27] Perry RJ, Non-invasive assessment of hepatic mitochondrial metabolism by positional isotopomer NMR tracer analysis (PINTA). Nature communications 8, 798 (2017). 10.1038/s41467-017-01143-w PMC5630596 28986525

[28] Sakuma I, Liver lipid droplet cholesterol content is a key determinant of metabolic dysfunctionassociated steatohepatitis. Proceedings of the National Academy of Sciences of the United States of America 122, e2502978122 (2025). 40310463 10.1073/pnas.2502978122 PMC12067271

[29] LaMoia TE, Cytosolic calcium regulates hepatic mitochondrial oxidation, intrahepatic lipolysis, and gluconeogenesis via CAMKII activation. Cell metabolism, (2024). 10.1016/j.cmet.2024.07.016 PMC11446666 39153480

[30] Perry RJ, Zhang D, Zhang XM, Boyer JL, Shulman GI. Controlled-release mitochondrial protonophore reverses diabetes and steatohepatitis in rats. Science 347, 1253–1256 (2015). 25721504 10.1126/science.aaa0672 PMC4495920

[31] Perry RJ, Reversal of hypertriglyceridemia, fatty liver disease, and insulin resistance by a livertargeted mitochondrial uncoupler. Cell metabolism 18, 740–748 (2013). 24206666 10.1016/j.cmet.2013.10.004 PMC4104686

[32] Samuel VT, Mechanism of hepatic insulin resistance in non-alcoholic fatty liver disease. The Journal of biological chemistry 279, 32345–32353 (2004). 15166226 10.1074/jbc.M313478200

[33] Goedeke L, Controlled-release mitochondrial protonophore (CRMP) reverses dyslipidemia and hepatic steatosis in dysmetabolic nonhuman primates. Science translational medicine 11, (2019). 10.1126/scitranslmed.aay0284 PMC6996238 31578240

[34] Yan C, FDXR drives primary and endocrine-resistant tumor cell growth in ER+ breast cancer via CPT1A-mediated fatty acid oxidation. Front Oncol 13, 1105117 (2023). 37207154 10.3389/fonc.2023.1105117 PMC10189134

[35] Slone JD, Integrated analysis of the molecular pathogenesis of FDXR-associated disease. Cell Death Dis 11, 423 (2020). 32499495 10.1038/s41419-020-2637-3 PMC7272433

[36] Shum M, Ngo J, Shirihai OS, Liesa M. Mitochondrial oxidative function in NAFLD: Friend or foe? Mol Metab 50, 101134 (2021). 33276146 10.1016/j.molmet.2020.101134 PMC8324685

[37] Yang L, Systemic Delivery of AAV-Fdxr Mitigates the Phenotypes of Mitochondrial Disorders in Fdxr Mutant Mice. Mol Ther Methods Clin Dev 18, 84–97 (2020). 32995353 10.1016/j.omtm.2020.05.021 PMC7488755

[38] Weinstein DA, Safety and Efficacy of DTX401, an AAV8-Mediated Liver-Directed Gene Therapy, in Adults With Glycogen Storage Disease Type I a (GSDIa). Journal of inherited metabolic disease 48, e70014 (2025). 40064185 10.1002/jimd.70014 PMC11893205

[39] Govaere O, Transcriptomic profiling across the nonalcoholic fatty liver disease spectrum reveals gene signatures for steatohepatitis and fibrosis. Science translational medicine 12, (2020). 10.1126/scitranslmed.aba4448 33268509

[40] Min-DeBartolo J, Thrombospondin-I is a critical modulator in non-alcoholic steatohepatitis (NASH). PloS one 14, e0226854 (2019). 31891606 10.1371/journal.pone.0226854 PMC6938381

[41] Ikeda K, Shiba S, Horie-Inoue K, Shimokata K, Inoue S. A stabilizing factor for mitochondrial respiratory supercomplex assembly regulates energy metabolism in muscle. Nature communications 4, 2147 (2013). 10.1038/ncomms3147 23857330

[42] Li X, Mechanisms by which adiponectin reverses high fat diet-induced insulin resistance in mice. Proceedings of the National Academy of Sciences of the United States of America, (2020). 10.1073/pnas.1922169117 PMC7768680 33293421

[43] Sakuma I, Lysophosphatidic acid triggers inflammation in the liver and white adipose tissue in rat models of 1-acyl-sn-glycerol-3-phosphate acyltransferase 2 deficiency and overnutrition. Proceedings of the National Academy of Sciences of the United States of America 120, e2312666120 (2023). 38127985 10.1073/pnas.2312666120 PMC10756285

